# Recalibrating Veterinary Medicine through Animal Welfare Science and Ethics for the 2020s

**DOI:** 10.3390/ani10040654

**Published:** 2020-04-09

**Authors:** Andreia De Paula Vieira, Raymond Anthony

**Affiliations:** 1 Faculty of Veterinary Medicine, School of Health Sciences, Universidade Positivo, R. Prof. Pedro V. P. De Souza, 5300, Curitiba 81280-330, Brazil; 2 Department of Philosophy, University of Alaska Anchorage, 3211 Providence Drive, Anchorage, AK 99508, USA

**Keywords:** animal welfare, animal health, veterinary ethics, One Health, welfare technology and systems, veterinary education

## Abstract

**Simple Summary:**

This article emphasizes the importance of educating veterinarians and veterinary students in animal welfare science and veterinary ethics, so that they can ably advance pertinent scientific knowledge and promote ethical thinking as trusted animal advocates in the 2020s. In light of this public expectation, a number of challenges are raised for veterinarians and the veterinary profession. These challenges involve: (1) re-envisioning the nature of disease treatment that goes beyond traditional conceptions of health or clinical matters, and which include animal welfare; (2) re-imagining disease prevention at the intersection of animal-human-ecosystem health; (3) developing core competencies in animal welfare science and ethics in order to provide professional leadership in animal welfare; and (4) taking a more active role in the development of novel networked devices, monitoring technologies and automated animal welfare solutions, and understanding their effects on the welfare of animals, human-animal relationships, and the veterinary profession in general.

**Abstract:**

What should leading discourses and innovation regarding animal welfare look like for the veterinary profession in the 2020s? This essay considers four main challenges into which veterinarians are increasingly being drawn, as they respond to increasing public expectation for them to be scientific and moral authorities in animal welfare in addition to their traditional role as trusted health experts. They include: (1) to go beyond traditional conceptions of health by adopting a holistic view that also considers animal welfare, not only disease treatment; (2) to reimagine their professional duties when it comes to disease prevention at the intersection of animal-human-ecosystem health; (3) to develop core competencies/proficiency in animal welfare science and ethics in order to navigate discourses concerning competing priorities and socio-political ideologies and to provide professional leadership in animal welfare; (4) to provide feedback on novel networked devices, monitoring technologies and automated animal welfare solutions and their impact on animals’ welfare. To competently navigate the intricacies of the socio-political and connected world as trusted authorities and conduits for innovation in and through animal welfare, veterinarians and veterinary students are encouraged to: (a) develop core competencies in veterinary ethics, animal welfare science and deliberative capacities that are well-informed by current multidisciplinary frameworks, such as One Health; (b) engage interested parties in more effective collaboration and ethical decision-making in order to address animal welfare related concerns within their immediate sphere of influence (e.g., in a given community); and (c) participate in the process of engineering and technological design that incorporates animals’ welfare data (such as their preferences) for real-time animal monitoring through adding animal scientific and values-aware evidence in information technology systems. In order to tackle these challenges, four pillars are suggested to help guide veterinarians and the veterinary profession. They are: Collaboration, Critical Engagement, Centeredness on Research, and Continuous Self-Critique.

## 1. Introduction

The article emphasizes the need for veterinarians and veterinary students to become proficient in animal welfare science and veterinary ethics. It emphasizes the importance of integrating animal welfare into veterinary medicine and discusses four main challenges to the veterinary profession in the 2020s. Briefly, veterinarians, as trusted medical experts, are increasingly being called on by the public and policy makers to also be scientific and moral authorities in animal welfare. They are: (1) expected to go beyond traditional conceptions of health by adopting a holistic view that also considers animal welfare, not only disease treatment; (2) challenged to reimagine their professional duties when it comes to disease prevention at the intersection of animal-human-ecosystem health; (3) increasingly forced to develop core competencies in animal welfare in order to provide professional leadership in animal welfare and navigate discourses concerning competing professional priorities and socio-political ideologies and; (4) asked to provide feedback on novel networked devices, monitoring technologies and automated animal welfare solutions, thus, requiring familiarity with cutting-edge engineering and technological developments in order to critically assess their application to animals’ welfare. 

These challenges go beyond veterinarians’ traditional technical or clinical expertise and should be supplemented by proficiency in veterinary ethics and animal welfare science. Also, in the 2020’s veterinarians will need to utilize their voice more as issues involving the moral status and welfare of animals become political and they are asked to provide informed leadership to society on responsible use and care of animals. In doing so, veterinarians will have to develop greater sensitivity to and knowledge of a broad range of scientific and ethical issues in veterinary practice. The article concludes by offering next-step suggestions about how veterinarians, as trusted medical and moral authorities on animal welfare science and ethics, can lead and participate in professional activities that result in better animal health and welfare outcomes. Here, four pillars are suggested to help guide veterinarians and the veterinary profession in tackling these challenges. They are: Collaboration, Critical Engagement, Centeredness on Research, and Continuous Self-Critique. 

The succeeding discussion draws on recent philosophical arguments, scientific animal welfare studies, and first-hand practical knowledge from working in professional veterinary settings.

## 2. Veterinary Medicine: Disease Treatment and Animal Welfare

Veterinarians tend to focus on treating diseases in their patients as their primary clinical responsibility. This focus is due in large part to a narrow conception of what matters to animals, namely, their health. Here, classic veterinary textbooks steer practitioners and students alike towards a commonly defined view of animal health that privileges biological functioning, homeostasis, productivity, and successful reproduction. However, this classical view is steadily being upended by current scientific evidence provided by animal welfare science that emphasizes the complexity of animals’ experiences and features of their environment [1,2,3]; both of which have relevance to clinical assessments of what matters to animals, at every step of clinical case management. The classical approach has also to contend with the values or ethical and scientific dimensions of what is in animals’ preferences [4]. Thus, taken together, a turn towards a broader conception of health to include the welfare of the animal [5,6]—one which is constituted by both animal welfare science and normative components [2,7]—is strongly recommended for contemporary veterinary medicine. 

With respect to the former scientific sense of what matters to animals, veterinarians should not consider themselves animal welfare specialists, unless they are also willing to consider and integrate (and not discount or dismiss) aspects of an animal’s welfare, such as their behavior and affective states and features of their husbandry or care and the relationship between the animal to its environment as part of their clinical assessments. Per their professional charge, veterinarians should be open to medical training and development of core competencies that embrace the more holistic conception of animal health [8] that includes animal welfare and truly gets to the best interests of their patients, clients and the various publics they serve. That is, they should be willing to contextualize health deficits within a larger and comprehensive understanding of public expectations about animals’ welfare. 

This more holistic approach to health has been described by a few veterinarians, such as Blood and Studdert [9,10]. They define animal health as “a state of physical and psychological well-being and of productivity including reproduction.” Similarly, Martin et al. [11] defines animal health holistically as “a state of complete physical, mental, and spiritual well-being” [12]. These definitions of animal health are consonant with the one used by the World Health Organization (WHO), which declared that “health is a state of complete physical, mental and social well-being and not merely the absence of disease or infirmity” (https://www.who.int/about/who-we-are/constitution). Noteworthy is that this more holistic definition of animal health has been employed by animal welfare scientists for a number of decades now, such as Broom [1], Fraser et al. [2], and Duncan [3], who see this expanded conception of health to be synonymous with “well-being” and “animal welfare.” These animal welfare scientists had clearly understood early on that considerations of animals’ welfare went beyond biological function, as commonly understood by traditional clinicians. Thus, veterinarians would benefit society enormously by broadening their view of animal health.

Furthermore (and with respect to the normative sense of what matters to animals), while the scientific study of animal welfare involves considerations regarding the qualities of animals, such as their behavior, health, affective states and physiology, animal welfare scientists were also some of the first to recognize that animal welfare is influenced by the attitudes and actions of humans toward animals [13,14]. This aspect of animal welfare reflects public attitudes about “what we owe” [15] to animals and a deeper acknowledgment of the relationship between animal welfare, environmental factors [16] and public health [17].

What we owe animals depends on an assessment of priorities of what is either good or bad for animals, through a framework that is informed by animal welfare science. Assessing these priorities involves making ethical decisions about what it is in the animal’s interest, which include being able to balance benefits and harms, evaluating the trade-offs among distinct scientific welfare indicators and judiciously reasoning through conflicting empirical claims. Animal welfare science can be characterized as the rigorous use of scientific methods to study the quality of life of animals, including that of companion animals, research animals, wildlife, and those farmed for food. This relatively new science (which has its inception in the 1960s) is borne out of ethical concern for animals [2,13] and can inform the choice of practices involving animals. Its focus is on investigating animal-based measures, including animals’ perspectives and preferences in a specific context. Animal welfare science develops and integrates behavioral, physiological, psychological, clinical and epidemiological measures and indicators of welfare to identify whether a specific context is either positive or negative for animals at individual or group levels [4,14]. Science-based interventions that promote animals’ positive affective states and expression of species-specific behaviors can inform interventions to prevent and treat diseases and vice-versa. In animal agriculture, for example, these interventions contribute to housing, handling, husbandry and management practices that can lead to good financial outcomes for producers, healthier and safer food for consumers and greater environmental sustainability.

For veterinarians practicing in the current decade, the upshot of a more holistic conception of health vis-à-vis animal welfare entails having solid grounding in both animal welfare science and veterinary (and including animal) ethics. Since animal welfare science is a relatively young field, veterinarians have a vital role to play in the development of research and funding opportunities that can strengthen its trajectory in response to societal questions about how we ought to treat animals. This includes calling for novel scientific investigations to inform place-based policies and guidelines.

Below, the article discusses how veterinarians may develop these competencies and raises some key challenges that the profession might encounter within this decade.

## 3. One Health: Disease Prevention and Animal Welfare

Animal welfare is a multidisciplinary concept [14] that challenges veterinarians to lead in establishing it into practice in ways that address its overlapping dimensions (i.e., affective states, biological functioning and ability to live according to species-specific behaviors and needs) and connection to public health and environmental factors. Veterinarians are increasingly being drawn into animal health and welfare issues that require them to demonstrate awareness of and professional leadership on multidisciplinary subjects at the intersection of human-animal-ecosystem health. In 2006, the American Medical Association and the American Veterinary Medical Association (AVMA) approved resolutions supporting the One Health initiative. A main driver of the 2006 One Health initiative was the increasing prevalence of serious disease outbreaks amongst both humans and animals around the world (see for example, https://www.cdc.gov/onehealth/index.html and https://www.nature.com/articles/nature06536#Fig1). The initiative “recognizes that human health (including mental health via the human-animal bond phenomenon), animal health, and ecosystem health are inextricably linked. One Health seeks to promote, improve and defend the health (and therefore well-being) of all species, by enhancing cooperation and collaboration between physicians, veterinarians, other scientific health and environmental professionals and by promoting strengths in leadership and management to achieve these goals” (http://www.onehealthinitiative.com/mission.php). The resolution marks the first time a holistic definition was formally agreed upon to address the interconnections between human-animal-ecosystem health, and it resulted in greater public visibility for the well-being of animals [18]. One Health considers the realities of the day, including the scaling up of agricultural production, housing billions of livestock in close and confined quarters, antimicrobial resistance, human encroachment into wilderness for development purposes, and exposure to new disease agents and vectors as human-animal interactions intensify. 

Accordingly, this ambitious initiative strongly encourages veterinary professionals to consider individual welfare concerns in the practice and take a holistic view when addressing issues related to the nexus of human-animal-ecosystem health. Here, the unique tripartite VCP relationship between veterinarians, clients and patients in the practice context, should be located within the broader context of public and environmental health. When first announced, the initiative was at odds with how most veterinarians were trained. Originally led by two physicians and a veterinarian working on epidemiological issues, the One Health initiative has since expanded, moving past discourses around antimicrobial resistance in which it has predominantly been featured in the past years [19]. In classical epidemiology, the typical orientation is toward biology and mechanisms of causation and populations and their interactions with the environment [20]. 

Under this construction, a disease emerges when an agent infects a host in some environment. Contemporary veterinary epidemiology, on the other hand, invites veterinarians to integrate quantitative and qualitative animal welfare science research for different species as a core component in epidemiological research and modelling to ensure better health and welfare of animals and protection of humans and the environment. This calls for a new set of approaches from epidemiological research to population studies. In contrast to classical epidemiology, epidemiological approaches that view animal welfare more holistically, should emphasize promoting optimum welfare as the basis of disease prevention – that is, by considering standard measures of physical health together with animal behavior and affective states and the impact of the complex relationships animals have with conspecifics, humans, the environment and other species (e.g., there is growing speculation that wildlife trafficking and/or conventional farming techniques may have contributed to the emergence of SARS 2 Covid-19 virus). 

Under the auspices of animal welfare science, “health” takes on new meaning, reflecting a wide range of multifactorial criteria that must be included in epidemiological models that influence disease occurrence and pathogeny. “Health” as defined by the WHO, is no longer only about the risk of an insect vector transmitting a direct causal agent to an animal during the rainy season, or promoting humane depopulation techniques for animals during epidemics. The 1948 WHO definition of health states that it is “a state of complete physical, mental and social well-being and not merely the absence of disease or infirmity.” Here, health is also about promoting an orientation that emphasizes intervention strategies that provide both humans and animals with safe environments that are protected against possible threats to their welfare (or health), in order to maximize their welfare. Thus, in the context of providing good animal care or husbandry for domesticated animals, veterinarians along with caregivers and owners have an acquired responsibility to ensure that these animals have an environment that maximizes positive affective states [3] and that they are able to “cope” by expressing species-specific natural behaviors [1], while minimizing the risk of diseases. Factors in the environment and those intrinsic to the human or animal are particularly salient under this framework. In the case of the animal, the nature of the exposure to the environment, experiences of good health and positive affective states and opportunities to express natural behaviors, as per Fraser et al. [2] most likely influence the animal’s clinical response to a certain agent, host or environment. In the clinical context, this entails that veterinarians should also consider how animals feel and behave as equally important to prevent diseases of epidemiological importance. 

For example, animals can be major reservoirs of zoonotic diseases that can be transmitted from animals to humans and vice versa. This is the case, especially when basic public health measures such as prevention, detection, monitoring and ending of outbreaks and epidemics through sanitation and epidemiological surveillance are not considered in our relationships with animals. Let us suppose that a group of people from a condo want to create a common area for pets to promote their welfare by designing welfare-friendly areas and promoting social encounters governed by welfare regulation, or that shopping malls want to welcome customers with pets by creating a common area for their pets and owners that meet welfare criteria but do not address public health issues. If not properly designed to meet animals’ needs or preferences and surveilled for public and animal health concerns, these areas may have the potential to become sites of distress for animals and zoonotic transmission. At the time of writing, the world is hostage to a new world pandemic (SARS 2 Covid-19), a spillover transmission that, by all accounts, surfaced at a live animal market in Hubei province in central China. Here, the reservoir hosts most likely transferred the virus either via an animal host before zoonotic transfer or via humans following zoonotic transfer [21]. Spillover transmissions, that consist of “successive processes that enable an animal pathogen to establish infection in a human,” result from interactions among factors, such as disease dynamics in the reservoir host, pathogen exposure and within-human factors [22]. They could become more common as our interactions with animals become increasingly intertwined with our interests in strengthening the human-animal bond. Thus, further research is needed to map how human activities, animal care and husbandry practices, both directly and indirectly, may influence disease transmission.

The current global SARS-2 Covid-19 pandemic reminds us that veterinarians are highly central professionals, since they are able to combine epidemiology, animal health, animal welfare science and ethics, and public and environmental health, to the benefit of animals and society. Hence, at the same time that humans struggle to fight pandemics worldwide, their relationships with animals increase and become more complex and humane, welfare measurements should be taken into account when formulating preventative measures and surveillance systems. Holistic frameworks such as the One Health framework can enable veterinarians to integrate both their clinical expertise in animal welfare with their public and environmental health responsibilities in order to obtain better outcomes for all. 

Multidisciplinary approaches, like the One Health initiative, also highlight the need for veterinarians to be cognizant of a wide swath of ethical responsibilities they have, not only to their patients and clients, but to their colleagues, the public at large, the profession and the environment. (For food animal veterinarians, for example, the responsibilities include collaboration for the optimal health of humans, animals, and the environment in animal agriculture. Their efforts will impact public health, help producers maintain their economic livelihoods and dignity through work and contribute to national and global food security through the production of safe, sustainable, nutritious and wholesome food. See, for example: https://www.onehealthcommission.org/index.cfm/38050/46766/bipartisan_one_health_congressional_bills_introduced_in_us_senate_and_house).

Increasing, veterinarians are looked upon to benefit society through their medical and scientific knowledge, practical experience and understanding of how animals may be benefited and harmed. Ensuring good animal welfare and meeting social expectations for the profession involves advising, reassuring and educating clients and the public, while fighting misinformation. In being conduits of information and in their capacity as animal advocates, veterinarians should inculcate ethical awareness and ethical knowledge of the professional responsibilities of veterinarians [23] so that they can facilitate client compliance and facilitate informed decision-making. What is acceptable or not, in order to ensure care for animal-human-environmental interests involves moral sensitivity to a plurality of public values, the ability to evaluate the merits of scientific animal welfare methods and a well-developed understanding of and skills to deliberate effectively about a broad range of ethical issues that impact the practice of upholding high standards of animal welfare as a clinician [5,15,24]. 

Veterinary ethics, which involves being informed of the values and ethical perspectives that shape conversations and decision-making around priorities and how they ought to be weighted in clinical practice, provides veterinarians with the tools for identifying and assessing core normative and empirical animal-human-environment components [25,26,27,28,29,30]. Furthermore, ethical reasoning skills can help shape how veterinarians manage their professional responsibilities and provide animal care and treat their clients, and how they assess the acceptability of treatment towards animals in general or discharge their duty to offer pertinent scientific knowledge and credible professional leadership when addressing animal welfare issues. Ultimately, good ethical decision-making that takes into consideration both a plurality of ways to care for animals respectfully and the constraints on their caregivers, can lead to better advocacy of and outcomes for animals [5,7,15].

An illustration of the relevant and vital intersection of veterinary epidemiology-ethics-animal welfare science may be instructive here. If not for animal welfare science and ethical concerns regarding the treatment of food animals motivating investigation, many livestock veterinarians today would probably still be recommending that dairy calves be housed individually to avoid disease transmission. Instead of housing calves individually, now we know, through controlled studies, that disease transmission can be prevented or mitigated through better colostrum management on farms. Furthermore, socially housed calves, when fed higher milk volumes via a teat and gradually weaned off milk to starter and forage, transition more smoothly to a solid diet, gain more weight and produce more milk [31,32,33]. These studies give veterinary epidemiologists a basis on which to perform future comparative studies aimed at improving dairy cattle husbandry and health. For example, in contrast to conventional practices that promote individual housing or restrict milk feeding, future studies could investigate the effects of feeding calves more milk or housing them in groups early on as a management strategy to minimize common husbandry issues. These studies may illuminate how diseases such as those that commonly occur during the transition period in dairy cows (3 weeks prior and 3 weeks after calving) can be minimized.

Incorporating animal welfare science and ethics into epidemiological studies could illuminate our current understanding of the natural history of a disease and of epidemic processes by considering characteristics of the agent, host and environment together with animal care and husbandry (e.g., success of immune transfer, epigenetic effects, level of pathogens in the environment, pre-clinical and clinical signs, local commitment to animal welfare, effects of the human-animal bond, ability to perform species-specific behaviors, and experience of positive and negative affect). Such characteristics could be used not only in observational and experimental studies, but also in predictive epidemiological models when deciding on criteria such as parsimony, goals and data “best fits.” It is paramount that current integrated models of epidemiological population projections (e.g., cohort component models, Bayesian probabilistic projections) begin to include current animal welfare science data and expert opinions in order to enhance our understanding of animals and how to improve their welfare in the short, middle and long term. These models would, for example, reflect cutting-edge animal welfare and health knowledge in disease outbreaks and overall animal welfare, thus allowing veterinary epidemiologists to better represent animals’ realities and coping mechanisms under professional frameworks within the One Health initiative. 

Lastly, how we frame the discussion of animal welfare [34] within the One Health framework influences both how veterinarians advocate for animals through the approach they take to formulate case management and the policy and practical recommendations they will make regarding how animals ought to be treated. From ethical cum scientific perspectives, this is significant, because it will involve questions of distributive justice vis-à-vis different models of resource allocation and conceptions of risk and ways benefits and harms are conceived and balanced [35]. It will also involve recognizing differing values and ethical viewpoints, arbitrating conflicts between the interest of patients, caregivers and the public, providing precise and credible advise on new scientific evidence about human-animal-ecosystem relationships, identifying and evaluating norms, practices and procedures that coincide with “what is owed to animals” [15], and developing skills of prioritizing, risk analysis, facilitating decision-making and effective communication, all of which contribute to a sense of professional identity for the veterinarian [7,26].

## 4. Veterinary Medicine and Animal Welfare: Shifting Priorities and Political Challenges

There are a number of challenges involved in inviting a reevaluation of the profession. *First*, *making wholesale cultural shifts in any profession is a monumental feat*, and any change will likely be gradual and take time. The norms within a profession like veterinary medicine are pluralistic, and thus, getting consensus among practitioners to embrace both animal welfare science and veterinary (and including animal) ethics as core competencies in their clinical approaches will not be easy. Preliminary steps to do so, however, are already afoot. For example, a case for their inclusion in veterinary curricula is being made across various institutions, couched in terms that reflect the profession’s continued commitment to uphold high standards of animal care in practice [23,36]. Veterinary institutions seeking to address and strengthen the link between animal welfare and various subject matters (e.g., epidemiology, food hygiene and zoonoses, animal management in disasters, and environmental management), and ultimately create a culture of being mindful of animal welfare throughout the curriculum, can look to recent examples for inspiration.

The year 2011, for example, marks a time when a model curriculum of animal welfare began to gain greater attention in veterinary curricula [36]. The Federation of Veterinarians of Europe (FVE), the Canadian Veterinary Medical Association (CVMA) and the AVMA, as part of a broader conception of the veterinarian’s professional “codes of ethics,” jointly highlighted the central responsibility of veterinarians in promoting animal welfare [24]. Furthermore, in 2011, the North American Veterinary Medical Education Consortium (NAVMEC) recognized the special role veterinarians play as intermediaries for animals and stewards of their interests and the urgency in defining and integrating animal welfare as a new area of emphasis for veterinary education. 

Veterinary colleges across the United States and Canada are currently in the midst of developing curriculum to enable veterinary students to acquire and master core competencies in animal welfare [37]. As part of its accreditation process, the AVMA Council on Education (see 80, Standard 7.9 (g)), which is partially responsible for veterinary college curricula in North America, requires that graduates possess “knowledge, skills, values, attitudes, aptitudes and behaviors necessary to address responsibly the health and well-being of animals in the context of ever-changing societal expectations.” These initiatives call for science-informed animal welfare competencies as a central element. 

Another example is the Ad Hoc Veterinary Education Group of the OIE. This group launched the recommendations on the competencies of graduating veterinarians [38] and in 2013 it recommended the veterinary education core curriculum [39]. In both documents, the OIE argued that knowledge and proficiency in animal welfare with a scientific basis should be a standard of excellence within the veterinary profession. Here, it is acknowledged that animal welfare includes evaluative, normative and social dimensions. 

*Second*, *society expects veterinarians to be animal advocates*. This entails proficiency in animal welfare. Here, animal welfare science is key to inform policies and best practices. Scientists and philosophers have also acknowledged that animal welfare is not only a scientific concept [34,40,41], for its roots are inherently normative [2,13]. It is sometimes referred to as a “mandated” science [14]. Since the concept of animal welfare is not value neutral, a fair amount of evaluative latitude may still need to be afforded to veterinarians when they make recommendations about what is best for an animal’s welfare. For example, should good welfare prioritize health above all other aspects of welfare? Or should welfare prioritize individual preferences or collective goods or take into consideration other public health and environmental concerns? 

*Third*, because of its normative dimensions, *animal welfare can also become a politicized concept*, as different factions jockey to claim control of the shape and direction of local agendas and priorities concerning how animals should be protected and cared for, what uses and treatment are morally correct and what constitutes a fair bargain for animals when humans benefit from them [42]. The success of animal advocacy groups and activists promoting an animal-rights-based agenda into mainstream consciousness through many different media channels and persuasion modes poses significant ethical challenges [43,44,45,46]. Some examples of animal rights activism include vilifying producers, scientists and institutions that use animals, spreading misinformation about animal use practices and distorting conceptualizations of animals and animal welfare, extremist actions to eliminate (what activists consider) “factory farming,” narrow conceptions of solidarity with sentient others [47], shaming consumers and producers of animal protein, and campaigning vociferously to “de-animalize” animal agriculture by creating “clean” or “synthetic” protein through biotechnological means, and thus, omitting the need for animals. While some animal activism has highlighted important shortcomings of animal production, such as overuse of antimicrobials and the pitfalls of resistance [27] and on-farm mistreatment of animals and welfare deficits, others have harmed opportunities to seek out answers that actually will improve the lives of animals. These include corrective measures for more humane systems through regulatory or structural reforms and collective or cooperative action that would incentivize behavioral change among major stakeholders who rely on animals. Animal activism that argues that no use of animals is ethically acceptable, ignores centuries of longstanding, complex and shared social, economic and ethical histories between humans and animals [48,49] that have resulted in good outcomes for animals [14].

In countries where citizens, veterinary professionals and students may have less familiarity with the pitfalls of certain popular framings of animals’ issues, e.g., the “animal welfare-animal rights concepts and debates,” or appreciate the subtle philosophical or scientific distinctions or ethical nuances, the language of animal rights and rhetorical arguments used solely to advocate for individual animals may resonate as a good idea at first. However, animal activism may actually harm animals, especially if it is lacking in both scientific credibility (e.g., inaccurate portrayal or obfuscation of what is animal welfare science, cherry picking or incomplete representation of welfare science data) and appreciation for more pragmatic framings of the issues (e.g., dismissing the complexities of how human-animal relationships are “blurred” or intertwined [49,50] or deploying blanket solutions that are uninformed by local stakeholder perspectives and knowledge). 

Animal activists can also polemicize debates concerning animal welfare. Polemicized debates frustrate more pragmatic deliberations that, while do not justify current impoverished conditions, are committed to reform-oriented remedies to improve the welfare of animals in existing systems. For example, time in the field with animal producers in largely agricultural communities often reveals that discussions of how to improve the welfare of animals in agricultural systems simultaneously intersect with a number of different factors. These factors include the economic structure of food production, how scientific controversies over how ‘animal welfare’ are conceived [34], day-to-day economic and moral constraints on producers [51,52], technological capacities of the local agricultural systems [48,50], and tradeoffs between improving animal welfare and benefits to environmental sustainability and planetary health [53,54,55]. Heavy and unfortunate focus on whether animals have rights or are moral persons (narrowly conceived) tends to overshadow inquiry into the welfare of animals and initiatives that can produce real-world solutions regarding how best to improve animals’ welfare [56]; see also https://speakingofresearch.com/about/). 

In the abovementioned contexts, veterinarians entering discourses involving animal welfare should be aware of contentious political conflicts that only stoke conflict and do not offer genuine solutions to remedy unnecessary and avoidable animal welfare deficits. In advocating for their patients and providing credible public advice, veterinary practitioners should help their clients and the public see past activist agendas that are not well informed by good animal welfare scientific evidence or are based on ethical arguments that are too narrow in scope.

*Fourth*, *regulations that politicize ‘animals’ rights,’ will ultimately result in greater suffering for both animals and humans*, if they do not consider the effects they might have on animal welfare and peoples’ capacities to implement them. For example, the ban on horse slaughter in the United States in 2007 is a case in point. This ban stems from changes in state regulations and riders included in federal appropriations laws, prohibiting the use of federal funds for food safety inspections of horse slaughter facilities. The legislation also banned the transport of live horses from the United States to countries where they could be slaughtered for human consumption [57]. The horse slaughter case raises questions about whether the moral authority of veterinarians (e.g., members of the American Association of Equine Practitioners (AAEP)) to amend the Horse Slaughter Act on animal welfare grounds resonates with the public and policy makers. While legislation has been introduced in the US Congress amending the Horse Protection Act and banning transport of horses to slaughter, this legislation has yet to be passed. As a consequence, thousands of horses continue to be neglected, have poor welfare and go hungry. Veterinarians who join discussions that emphasize animal rights over other deliberative frameworks (e.g., pragmatic ones) should be aware that these discussions may be framed by activism for animal causes that are absent of or misrepresent important scientific parameters that reflect animals’ preferences (e.g., neglect to contextualize animal-based measures vis-à-vis a comprehensive understanding of animal welfare or overstate the extent to which the public or animal caretakers are motivated to change their habits due to animal welfare issues or economic interests) [14]. These discussions may also ignore the moral relevance of human-animal-environment relationships [58] or the substantial literature in animal welfare science (e.g., *Applied Animal Behaviour Science*, *Animals*, *Animal Welfare*, *Journal of Applied Animal Welfare Science, Frontiers in Veterinary Science*) that seek to improve the circumstances of animals within the context of improved technology, management practices, housing and animal care.

In giving voice to animal issues, veterinarians are vital in promoting a more nuanced and thoroughgoing account of an issue. As seemingly ‘anointed’ exponents of animals, the public may require credible and “honest” adjudication of the issues [59] from veterinarians. In some cases, veterinarians need additional training (e.g., ethical skills to identify veterinary norms, develop ethical reasoning and ability to recall animal welfare science and regulations, and develop a professional identity to advocate for animals’ welfare) to be considered credible exponents for animal welfare. In other cases, they may need to learn how to utilize their voice more in order to advance the societal role of the veterinary profession. Here, veterinarians may be ethically obliged to facilitate conscientious dialogue, for diverse publics, on the acceptability of certain practices towards animals. When doing so, they should be able to recognize and consider a plurality of values and viewpoints, reflect upon their implicit biases, and provide reliable empirical and value-aware evidence. 

In facilitating conscientious dialogue on animals issues, veterinarians should speak thoughtfully to how ethics and welfare science should be integrated into clinical practice and management decisions, and how the marriage of science-ethics can inform decision-making on public norms or enforceable regulations. This is particularly urgent in the emotionally charged context of animal cruelty, abuse and neglect, where giving an accurate account of an animal’s welfare is paramount. In some cases, zealous law makers or regulators may enforce blanket policies or fines that go against the recommendations of or do not take into account animal welfare science. 

Research funding and implementation of science informed practices will be contingent on the likelihood that the proposed design meets expectations of social, environmental and economic sustainability. In the agricultural context, for example, during an emergency, both normative and scientific dimensions of animal welfare should be considered and mapped out in contingency and action plans to minimize animal suffering and loss of life. The consequence for farmers, for example, when animal issues are not discussed with an adequate understanding of the appropriate science and husbandry, economic and technological constraints, are solutions that may not improve their animals’ lives, are not practical for producers to implement, and do not resonate with widespread public values regarding the permissibility of raising animals for food. 

Excluding broader stakeholder input and the imposition of lousy legislation or untutored policies can lead veterinarians and other exponents of animals to be at odds with their professional commitments [60,61]. They can also potentially disrupt meaningful discourses around animal use and care and ultimately fracture healthy human-animal interactions [62]. In other cases, they can undermine the role of veterinarians in advocating for animals or promoting their welfare [63].

In a climate where animal issues are increasingly politicized, the discussion above strongly suggests that veterinarians should be wary of merely capitulating to animal activist groups. Instead, they should take into account the mixed pressures on them or the profession when ensuring that animal welfare is prioritized as much as possible in the given context [18,64]. They may resist the pull of activism by advocating for and being proficient in animal welfare science and ethics. Here, partnering in multidisciplinary teams made up of veterinarians, animal welfare scientists and others trained in animal and veterinary ethics and/or who have practical experience with animal care, can help veterinarians in their capacity as trusted animal advocates and health experts. Such partnerships can provide enlightened public and policy-maker support to better understand animal value chains, and pinpoint where gaps may lie at the local level, in order to come up with creative animal welfare solutions through scientific, technological, medical and policy innovations (for example, see [65]). 

Furthermore, veterinarians can provide leadership by exciting policymakers, producers, consumers, retailers and industry agents to make the interests of animals a priority by funding animal welfare science that supports conscientious decision-making processes and studies of local food systems within a multidisciplinary context or public interest framework. The leadership of veterinarians is central when going beyond animal welfare benchmarking/assessment/standards, by focusing on criteria that include correlations with scientifically validated on-farm welfare measurements that reflect animals’ contexts and environments. The significance of funding agencies to finance local animal welfare science and its application in animal systems (e.g., food, lab, wildlife, companion) in humane and socially just ways will enable animals, human communities and the environments where they live to flourish. As trusted exponents of animals with special expertise and social clout, veterinarians can enable shared value initiatives, promote better communication through local scientific development and judicious use of local laws, and encourage the development of appropriate technologies to promote animal welfare, positive human-animal interactions and robust pathways in the information age with animal welfare science at its core.

## 5. Should all Veterinarians be Animal Welfare Experts?

At this stage of the article, a number of questions can be raised against one of the article’s primary theses. Should all veterinarians be animal welfare experts or scientists? Or should the profession promote specialization in animal welfare? How should the veterinary profession advance animal welfare knowledge for the benefit of society and veterinary medicine? 

To a large extent, forces beyond the profession are increasingly ushering this new dawn for veterinarians. Veterinarians are expected to provide their time, talent and training on how to manage and respond to disasters, zoonotic diseases and pandemics. They are also expected to play a role in assessing animal welfare risk factors and develop models beyond traditional views of health and economics for the care of companion animals, livestock, laboratory and research animals, and wildlife.

As trusted sources of knowledge regarding animal health and welfare and (increasingly so) moral authorities on behalf of animals, it is paramount that veterinarians build on the holistic framework that the profession as a whole has publicly agreed to be part of. The diverse ways in which animals interact with human individuals and within human communities and environments emphasize the need for new thinking regarding how to listen to animals’ perspectives and how to promote their welfare interests within a multi-stakeholder and multidisciplinary context. 

Multidisciplinary initiatives, like One Health previously discussed, invites veterinarians to recognize both the distinct nature or animal welfare and its multifaceted nature—animal welfare science, clinical and population medicine and ethics. It encourages veterinarians to play a more active role in discourses surrounding animal welfare issues, challenges and questions. By integrating their expertise in animal health and biology with evidenced-based findings, regarding the behavior and psychology of animals and society’s concerns about animals, veterinarians (and the profession for that matter) will be better equipped to provide broader and trusted leadership and advice around both medical and moral issues related to the health and well-being of animals. Sample questions in this regard include: How do animals respond to contemporary facilities with and without effective animal husbandry? What is their natural behavior and what does it mean for the context in which they are living? What is their subjective experience of pain and pleasure? What are their preferences? What are the best measures of health and coping? How does animal welfare prevent disease dissemination and pathogenicity? How do changes in the environment according to animal welfare principles can improve veterinary practice? How can animal practices prevent common diseases observed in systems of poor welfare? What priority should be given to welfare science in the formulation of laws and regulations to ameliorate local conflicts between humans and animals? 

In animal agriculture, innovative thinking by veterinarians is especially urgent given the pressing need to be judicious about using antimicrobials [27,29], especially when little is known about the effects of positive affective states on how well animals cope immunologically to infections. For example, by being proficient in animal welfare science and animal husbandry, could veterinarians demonstrate the benefits of improved welfare practices in reducing the spread of infectious diseases (and zoonoses) among animals, by boosting animals’ immunity through reduced negative affective states or the promotion of positive ones? This would not only improve animal welfare, but could potentially reduce the need for therapeutic antimicrobial usage, for example. 

Considering another example, when veterinarians engage in conservation initiatives involving wildlife, innovative thinking by veterinarians is urgent, not only to understand how to stem infectious diseases or zoonotic transfer when humans encroach on wild populations, species and ecosystems, but also by improving positive outcomes for wildlife vis-a-vis human-animal encounters or displacement. Veterinarians trained in animal behavior and welfare are central to the care of wildlife since they hold not only knowledge in wildlife health, population medicine and environmental management. They are also able to maximize wildlife welfare during rescue, rehabilitation, release and monitoring and during captivity by providing the animals good health care and more enriched environments. The same goes for laboratory animals. Veterinarians are essential in the development of novel technological solutions to monitor animals remotely in order to ensure that these animals have environments and husbandry that meet their preferences and needs. Doing so helps to ensure that laboratory animals and their handlers continue to be safe and that these animals remain suitable experimental models. 

As veterinarians think about how their expertise may be better utilized in the future and ways to integrate the confluence of clinical-social-ethical challenges to the professions, innovating in and through animal welfare calls on veterinarians to look beyond traditional veterinary medicine discourses or solutions. It requires that veterinarians consider animal welfare as a significant aspect of veterinary medicine, by addressing the multi-factorial dimensions of animal welfare. This includes making important value and conceptual judgements about what constitutes good welfare for animals and how to assess it scientifically [7,34]. When addressing zoonotic diseases in an One Health approach, for example, veterinarians should highlight systems thinking and collaborate with experts from different professional disciplines who can lend insight into the roles animals play in promoting healthy and sustainable human-animal-ecosystems and on how external factors (e.g., husbandry, economics and environment) may shape animals’ lives. 

Challenges are aplenty, however, and may frustrate veterinarians wishing to engage with those outside their profession or those already trained in animal welfare. For example, the human medical community has been slow to engage in ethical discourse around zoonoses. Through a case study of the 2014 Ebola outbreak in western Africa, Thompson and List [66] highlight that early responses by the human medical community to address the issue seemed lukewarm and disconnected from veterinary research on cross-species contagion and the virus’s connections with animal populations (see also [67]). The same authors also highlight a failure to engage with environmental ethical concerns, including those related to the moral status of wildlife (e.g., bats, gorillas and chimpanzees) and domestic animals (dogs) whose welfare was directly and indirectly impacted by the outbreak (see also [68,69,70]. Furthermore, attention to the role of climate change and sustainability issues did not emerge until the end of the outbreak [66]. 

Thompson and List [66] and Lapinski et al. [71] challenge all medical professionals engaged in the One Health initiative to develop inclusive assessment and ethical frameworks that complement the current focus on technical considerations or clinical problems, zoonotic diseases, and include connections to human, social-cultural, practical and policy concerns, as well as to health infrastructure that are under the scientific framework of animal welfare and the social sciences. In doing so, veterinarians and human medical practitioners have an opportunity to reflect on their moral beliefs (and how these beliefs motivate action), social and cultural biases, and the pervasiveness of ‘groupthink’ in framing ethical issues. Furthermore, it allows veterinary practitioners to consider global-level distributive, recognition and social justice issues, such as resource allocation, risk sharing and interspecific and intergenerational justice, in addressing public and international One Health issues [47,72,73].

Moreover, Verweij and Bovernkerk [74] raise questions about how the One Health framework can unite medical doctors and veterinarians in their cooperation. Their concerns are categorized into issues of scope, alignment and vagueness of the concept. In terms of scope, while the concept “is first and foremost a practical and programmatic call for interdisciplinary collaboration,” the concept may be too much of a catchall to be effective for on-the-ground study of causal interconnections between interspecies and ecosystem function. In terms of alignment, the two metaphysical assumptions, i.e., of “harmony or synergy” across different health systems (that there exists “an overall health state” of an ecosystem), and the belief that both human and non-human entities (including environmental systems and constituents) have certain “moral status,” which is action-guiding when it comes to the choice of interventions, is not uncontroversial. Where vagueness is concerned, veterinarians have an important role to play in shepherding discussions about the different meanings of One Health and in teasing out the metaphysical, analytical and ethical components of the concept for both policy development and practice (see also [66,75]). 

Veterinarians also have a crucial role to play in enhancing medical decision-making frameworks and ethical deliberation, since they may bring different substantive concerns and perspectives on how to arbitrate trade-offs between animal-human-environment conflicts. For example, veterinarians will bring their clinical experiences and perspectives regarding animal health, biological functioning, affective states, distress and behavior and professional view of respect and care towards animal life to address topics such as prophylactic depopulation or emergency measures to protect uninfected animals and the livelihoods of farmers, or limiting antimicrobial usage to promote good outcomes for animals, the public health, future populations and the environment (fewer pollutants and less residue) [65,76,77]. Central to these deliberations will be an acumen to recognize and identify key ethical concerns around questionable practices and procedures in veterinary medicine. Frameworks like the One Health approach give veterinarians the opportunity to clarify their values and motivations and potentially support consensus building where there is conflict. It also allows veterinarians to work methodically through a series of questions including (adapted from [78]): What is the harm? Who are the stakeholders? How are the harms and benefits distributed? What information do we have? What information should we know before making the decision? What are the options? What ethical principles should guide us? Have the relevant stakeholders been invited to participate in decision-making or have their views represented? How should moral closure be established?

## 6. Veterinary Medicine, Animal Welfare and Precision Monitoring Technologies (PMTs)

The advent of precision monitoring technologies (PMTs) together with heightened computer processing power and sensor technologies have provided companion animal caregivers, agriculture and food industries with the ability to collect and store large amounts of data on animals. Precision animal monitoring devices are essentially networked technologies that continuously monitor and manage individual animals automatically via algorithms and compare this information to expected production, behavioral and animal welfare benchmarks [65,79]. In the case of animal agriculture, for example, precision livestock farming (PLF) data can be used to improve the efficiency and sustainability of various animal industries [80] and hold the promise of improved animal welfare (e.g., [81,82,83]). Animals are reduced to (and further commodified into) CTI systems, namely, “Complex, Individual, and Time Variants” [84]. 

While not the goal of this paper, it is prudent to note that there are foreseeable sensitive ethical problems about the animals that will develop from employing these technologies in the field, especially related to who owns the data: the farmer or the developer? How can the data be used and by whom, for what end? How transparent to society should the data obtained from animals be? How is farmer autonomy or identity dealt with? For companion animals, if they are designed with animal needs in mind, do the technologies offer the potential for caregivers to know more about the lives of their animals, especially when they are apart? To date, however, many technology developers still do not include veterinarians specializing in animal welfare or animal welfare specialists as part of a development team. Including these practitioners and welfare scientists are important since they may know how a device or piece of equipment could harm animals during prototyping and testing. Since animals cannot speak verbally, the special training possessed by these veterinarians and welfare scientists allow for needed interpretation of animals’ preferences, behavior, health and affective states during prototype testing. Furthermore, a specialist in animal welfare science is also important in defining what scientific welfare parameters to consider when interpreting raw data gathered from monitoring systems (e.g., behavioral, psychological or pathological welfare indicators). Perhaps one way of protecting animals is to make veterinarians, working in tandem with IT developers and regulators, technically responsible for the animals in the development of processes and products. This would ensure that devices and data-systems are developed with animals’ preferences in mind.

Practically, a major challenge for veterinarians is the extent to which the technology will relieve them of their professional duties to be attentive to individual animals. These technologies will allow for real-time surveillance and monitoring and standardized measures of welfare, timely detection of poor welfare or illness in order to minimize treatment costs. Developers often extol the benefits of minimal human interaction with certain animals as a plus of these technologies. However, this displacement of manual attention and care already poses an ethical challenge for farmers [65,79], but at the same time may help animals in places where there are unqualified workers. Also, veterinarians would be required to have the requisite training to properly use or interface with the technology, interpret the continuous stream of data and apply it in a way that promotes animal welfare and positive human-animal relationships. Some knowledge in computer programming would benefit the veterinarian. Another concern involves making sure that these technologies do not take the place of good husbandry, animal care or conceal ways in which animals communicate their needs and preference to their caregivers [65,79,85]. From an ethical perspective, it is vital that such technologies do not relegate animals further to the status of undifferentiated commodities, unfamiliar to the caregivers as sentient beings with rich psychological experiences. Veterinarians have a role to play in offering guidance in the development of technologies that could otherwise diminish the benefits of ages of domestication of animals that have proven vital to the human-animal bond. 

How might veterinarians use the technologies to better understand the point of view of the animal (i.e., to interpret animal-based measures that reflect the preferences and needs of an animal)? How can robust welfare indicators be developed and included in computational algorithms that allow for machine learning to be applied in a wide range of settings where animals are involved? With PMT devices becoming more prevalent in households and on farms, having competence in understanding the data from PMTs will be particularly vital for veterinarians if the data can be interpreted to help promote better animal welfare and human-animal interactions. 

Veterinarians are central advocates for the welfare of animals [24,86], and accordingly it is important that they be able to comment proficiently on the place of these devices in improving welfare as well as guide programmers and animal owners on how best to use these technologies to promote healthier human-animal-environment interactions according to the best available scientific evidence. While technology designers, programmers or commercial agents, for example, can speak about animal welfare or claim to know what is in the best interests of animals, the public still looks to veterinarians to protect and promote the interests of animals. Hence, there is a need for veterinarians to respond to this public expectation to guide animal-related technological innovation, scientifically and ethically. 

Veterinarians with specialization in animal welfare should take part in the product design or software development, so that the perspective of the end-user (i.e., animal) is modelled or evidenced on the final product or system together with those who care for them. Usability tests and animal factors should take the perspective of the animal into account and this is done by drawing on animal welfare science methods, not just design or engineering methods. For this reason, veterinarians need to be accomplished and proficient in all the dimensions of animal welfare, including user experience techniques, in order not to lose their position of leadership and trusted authority when defending animals’ best interests during product development.

Whether it be in livestock farming, biomedical research or clinical settings, in shelters or in the wild, veterinarians will be expected to know how to use these technological tools, to interpret the data, relevant scientific studies and animal responses, in order to meet the natural proclivities of animals of a given species. They will also be asked to provide guidance on animals’ medical conditions, emotional and cognitive well-being and behavior during the animals’ interactions with their surrounding environment, with different technologies and with human beings in various contexts. This will require careful understanding of PMTs, familiarity with statistics, IT project development, computer programming, data visualization, machine learning and prototyping in order to apply the data to understand animals’ preferences, motivations and needs (see, for example, [80,81]). Knowledge of animal welfare science will be crucial for veterinarians to know the impact of these technologies on animals [62,80,81]. Thus, veterinarians need additional education and training in animal welfare so that they may adequately be exponents on animal welfare issues. It is worth noting that if a veterinarian has little or no training in animal welfare science to help him/her contextualize the animal-technology-environment-husbandry interface, then he/she could be performing a loose examination of the welfare of the animal in the given case. This is likely if the veterinarian merely appeals to off-the-shelf welfare standards to support his/her animal welfare arguments (especially if he/she lacks scientific contextualization of the specific reality under which the animal is placed). 

As veterinarians become more familiar with animal welfare science, animal behavior, PMTs, and marry their expertise in veterinary health, disease, food quality, zoonoses prevention and mitigation, and epidemiology, more profound elucidations on the perspectives of animals within their environments and the effects of the environment on them can be made. This can help improve the welfare of animals, by contextualizing their health as well as their physical, behavioral and mental states. For example, through behavioral function, phylogeny, mechanism and ontogeny, experimental psychology and design, systems management, epidemiology, stress physiology, animal-computer interactions and veterinary medicine practice, veterinarians can better understand the interests of animals [1,2,3,12,14]. 

Veterinarians, animal scientists and other scholars working on animal welfare issues can help innovate and change difficult realities for both humans and animals. When applied to animal agriculture, for example, veterinarians, as part of multidisciplinary teams, can work to precisely map the local demands of each aspect of the chain, balancing the needs of all stakeholders, not merely large agribusinesses. Here, multidisciplinary teams who marry animal welfare science, indicators and values can innovate certification programs, retailer practices, communication strategies, business benchmarking, information technology and so on, and bring greater transparency to the public so that they may grasp what is happening at the farm level, to make better informed decisions. Through platforms of knowledge, topics the public cares about can also be made available relatively easily and promote transparency to society. 

Internet of Things (IoT) systems can be deployed to monitor animals through sensors that reveal variables such as animal behavior, weight and location, which informs caretakers about their welfare in real time. Common constrains related to IoT systems (especially when applied in remote areas) include storage restrictions, accessibility to infrastructure, piracy and security issues, internet quality and bandwidth capacity. Moreover, many of the scientifically validated welfare applications available today, such as the Horse Grimace Scale and Welgoat Apps developed by the Animal Welfare Indicators Network, could be connected to online species-specific assessment protocols, e-government platforms, databanks, analytics, interactive media and be used by well-trained welfare assessors. Knowing what animals need or want and becoming familiar with their natures is essential to the sustainability and health of human-animal-ecological systems in agriculture. An ethical policy regarding technologies to adopt should focus on all aspects of the design and development process, not just the end products. Greater emphasis should be placed on upstream interventions focusing on a variety of determinants of welfare, as well as the environmental factors that contribute and sustain positive human-animal interactions and relationships.

## 7. Veterinary Medicine, Animal Welfare and Education

The challenges discussed above call veterinarians to increase their specialized competencies in animal welfare education and training. For example, the *OIE Recommendations on the Competencies of Graduating Veterinarians (‘Day 1 Graduates’) to Assure National Veterinary Services of Quality* (https://www.oie.int/fileadmin/Home/eng/Support_to_OIE_Members/Vet_Edu_AHG/DAY_1/DAYONE-B-ang-vC.pdf) and the *AVMA’s One Health Recommendations* (https://www.avma.org/KB/Resources/Reports/Pages/One-Health41.aspx) underscore the need for veterinarians to have proficiency in animal welfare science and ethics in order to be the leading advocates for the welfare of animals. These important recommendations also stress that veterinarians should develop the capacity to evaluate and solve clinical welfare concerns within multidisciplinary and rapidly changing contexts.

Recent global events involving human-animal-ecosystem interactions (e.g., Covid-19, Avian Flu, Ebola, Zika, global food security, invasive species, pandemics and zoonoses involving depopulation) punctuate this point and have necessitated multidisciplinary frameworks such as the One Health framework and teams to address them. In Europe, welfare indicators and protocols have been developed to promote the sustainability of different animal systems. In the United States, the AVMA has been developing guidelines for euthanasia (since the 1990s, most recently updated in 2020 [87]), humane slaughter (first published in 2016), and depopulation (first published in 2019). The process of constructing guidelines has relied on multi-disciplinary teams, which includes veterinarians representing various species, animal welfare scientists and an ethicist, to determine the best possible techniques for humanely terminating animals under different conditions. In Canada, codes of practices (e.g., NFACC—http://www.nfacc.ca/) have been developed within multidisciplinary settings, largely informed by the best available animal welfare science and reflective of social values. Private companies and labeling enterprises have also been benchmarking for animal welfare. 

With appropriate training in the scientific, ethical (e.g., the intersection of human-animal-environmental ethics) and social aspects of animal welfare and an appreciation for PMTs, the involvement of veterinarians can ensure that more attention is paid to monitoring the welfare and health of animals on-farm, commensurate with place-based needs and in real-time. Furthermore, as an organized profession and in partnership with scientists and other experts, veterinarians can help to make clear(er), and give visibility to, initiatives, like the ones highlighted in this article, that reflect their values and commitments towards animals and their welfare.

Since animal welfare has normative and scientific components [2], familiarity with animal welfare concepts also involves being more aware of the effects on individual animals and populations. It also means acquiring skills in ethical reasoning and animal welfare science methods so that veterinarians can better engage with a variety of stakeholders. Acquiring these competencies can help veterinarians consider ways to integrate ethical reflection into practical or clinical settings and improve ethics dialogue, both within the profession and with external stakeholders (see, for example, [60,61,88]). In doing so, veterinarians will be in a better position to engage with the sciences and the different publics they encounter when disentangling dilemmas associated with animal health, behavior and affective states. Being proficient in these areas will enable veterinarians to lead conversations about the development of standards and regulations involving animal use in areas such as animal agriculture and biomedical research. Furthermore, it allows them to provide consistent and measured guidance to their clients, regulators and industry agents responsible for enforcing voluntary and mandated welfare and health requirements as well as innovate in areas such as human and environmental health and science. For ethicists working with veterinarians, these challenges can stimulate consideration of novel deliberative frameworks to tackle the normative dimensions of pressing empirical and practical matters, potentially strengthening the philosophical basis of bioethics and normative ethics. 

PMTs challenge current and future generations of veterinarians to think conscientiously about the perspectives of animals in tandem with their other professional responsibilities (including ethical and scientific ones) to the profession and the public [83]. They challenge veterinarians to be cognizant about better husbandry possibilities made possible through technology and science (e.g., [89]) and to improve communication possibilities with animals (enhance the ways animals inform us about how they are faring) and for animals to “improve their participation in their lives” within contexts such as industrial farming [90]. Acquiring competencies in animal welfare science and ethics can enable veterinarians to assist their clients with contextualizing PMT-generated data and further inform and transform multidisciplinary frameworks to address real-world concerns, ranging from dealing with zoonotic diseases to producing a higher quality and sustainable product. A high degree of competency in animal welfare will enable veterinarians to deal judiciously with scientific facts and values issues that influence the interests of their clients, the profession itself and, most importantly, the animals.

With One Health and PMTs permeating the medical and veterinary landscape - and as the public becomes increasingly sophisticated in their view of the moral status of animals, what they can know about the inner lives of animals and how we raise and use animals globally - the effort to revise veterinary curricula should be done so that training and education in animal welfare is addressed consistently and inclusively [91]. How pedagogy should be expressed, and what scientific, technological and governance-related innovations should be developed and articulated carefully to address both global and place-based concerns, will also be key moving forward. More opportunities, such as the OIE’s Global Conference on Veterinary Education, the International Conference on the Assessment of Animal Welfare at Farm and Group Level, or the Conference on Animal-Computer Interaction and through the Animal Welfare Science Hub, should be encouraged to bring educators and animal welfare researchers from across the globe to brainstorm effective ways to promote animal welfare education and research in veterinary curriculum. Such initiatives can also serve to identify ways to overcome hurdles in and resistance toward integrating animal welfare content into existing veterinary curricula. Finding ways to effectively evaluate animal welfare competencies, the knowledge of new veterinary graduates and existing practitioners [36,92] and encouraging veterinarians to be conduits for animal welfare will be essential in addressing global issues, such as food security and the sustainability and health of our communities.

As multidisciplinary frameworks, like One Health, gain more currency and urgency amongst veterinarians, integrating knowledge about the welfare of animals—not merely for the benefit of the animals but also for ecosystem and human health—puts veterinarians in a position to be credible advocates for and honest brokers in promoting the interests of animals and increasing public trust [7,8,23,93]. That is, for veterinarians to competently provide sound advice on animal welfare issues and help their clients and the public make informed decisions, veterinary schools should train their graduates to possess both knowledge and skills in animal welfare science so that they are able to use their training to evaluate complex ethical dilemmas involving animals, people and the environment. 

## 8. Next Steps

While developing expertise in animal welfare may not be the bread and butter of all veterinarians at the moment, proficiency in some central aspects of animal welfare science and ethics will likely be essential. Given the time constraints of working in veterinary practice, web-based continuing education courses or symposia dedicated to animal welfare issues (e.g., AVMA’s Animal Welfare track at the annual convention) are examples of low-hanging options.

Furthermore, successful responses to emerging challenges for veterinarians as trusted authorities of animal welfare will not only be a scientific or clinical undertaking, it will be an ethical undertaking. This means that veterinary strategies to overcome the challenges identified herein need to be informed and guided by ethical analysis that is supported by the most current animal welfare science. As we have demonstrated, the three main challenges will inevitably involve ethical implications in the decision-making process.

Veterinarians, given their professional expertise and the public expectation of veterinary professionals, have an opportunity to play a more active role in engaging with a broader set of stakeholders in multidisciplinary settings. Veterinarians offer a unique perspective as trusted advocates for animals through scientific inquiry, ethical deliberation, innovative and current communication technologies and media, and multidisciplinary frameworks that place animal welfare as a central catalyst for the health and sustainability of a human-animal-ecological system. 

As advocates of animals and advisors to clients and the public, how veterinarians approach the profession and epidemiological studies, as well as how they encourage veterinary medicine and animal health to evolve concurrently with science, technology and ethics, will be essential in promoting healthy debates around the intersectionality of human-animal-environment. Work in this area is much needed as we attempt to find ethically and science-informed solutions to complex global challenges. 

The global veterinary community faces many challenges as the profession attempts to innovate. Central among these include the following: How training in animal welfare should be implemented in the veterinary curriculum at local levels? How to deal with crowded curricula [36]? How to effectively overcome the lack of local or place-based science addressing animal welfare, and human or public health and environmental issues [29,30,94], and the dearth of well-trained animal welfare specialists? How to manage impediments in developing appropriate instructional resources and defining curriculum objectives? How to handle the inability of veterinary organizations to delineate key and shared competencies in animal welfare and to distill best practices in assessing animal welfare? 

Overcoming these challenges will be necessary to meet global One Health threats involving animals and to understand changing social expectations towards the profession and public sentiment regarding the moral significance and acceptability of practices towards animals worldwide. The following underlying pillars may help guide the veterinary profession in tackling these challenges: Collaboration, Critical Engagement, Centeredness on Research, and Continuous Self-Critique. 

*Collaboration*

The impulse for collaboration acknowledges the multidisciplinary dimensions of diagnosing contemporary problems and the necessity to engage non-veterinarians (e.g., animal scientists, ethicists, medical practitioners, IT specialists, designers, environmental scientists) to problem-solve and promote objectivity if prescriptions are made. The multifactorial nature of animal welfare lends itself to interesting cross-pollinations and growth for this area of study. For example, collaboration between veterinarians working on animal welfare issues and different branches of bioethics (e.g., animal and veterinary ethics, public health ethics, environmental and agricultural ethics, philosophy of technology) is also desirable to meet the human medical, public health, technological, and environmental challenges of the future. The veterinary profession has become increasingly specialized, with veterinarians focusing on their own subject matter. These challenges and how they intersect with the welfare of the different species (from both scientific and ethical standpoints) can facilitate much needed interdisciplinary connections beyond a focus on traditional clinical practice. They encourage veterinarians to broaden their understanding of the normative and scientific questions facing their profession, the political and moral dimensions of their work, both locally and globally, and the cultural dimensions involved in addressing collective action problems such as zoonoses and the role that welfare may have in preventing them. Veterinarians working in the areas of animal welfare can benefit from exploring the possibilities of using the normative and conceptual analyses from other disciplines, including ethics and political, social environmental and public health sciences [60,64]. For example, as noted by [74], “work in public health ethics on how to understand the conceptual relation between individual health and collective health, can be fruitful for animal and environmental ethics, where there is often a polarized debate between scholars about the moral status of animals” (p. 2). 

*Critical Engagement*

Critical engagement with others (e.g., in the normative and social sciences) instills an openness to dialogue with goals to appreciate the pre-commitments of their profession and the individuals who constitute it, as well as the perspective of the animal as a central stakeholder and the tensions inherent both within the concept of “animal welfare” (i.e., how to balance an animal’s need to express natural behavior with its preferences and affective states, and physiological measures) and from without (i.e., how to consider both human and non-human perspectives and values). It also encourages a deeper commitment to problem identification from various perspectives, especially less dominant ones, so that all the relevant facts and a variety of alternatives can be shared and considered [95]. As major professional advocates for the interests of animals, veterinarians are essential to multidisciplinary teams tasked with addressing global concerns related to the health of human-animal-ecological systems. Their engagement in these teams is essential to facilitate resolving place-based dilemmas with competence, credibility and fairness.

*Centeredness on Research*

A centeredness on research highlights the importance of continuous innovation and scientific excellence. Here, innovation in veterinary medicine can occur best (i) through and in animal welfare science, the field of inquiry specifically committed to probing the interests of animals from their perspectives and their ‘local realities,’ in tandem with fruitful collaborations with non-veterinarians to consider the best deliberative frameworks from which to develop standards of best practice, and consider a broader view of ‘the relevant literature,’ modes of training and instructional design; and (ii) developing competencies in good governance and risk communication to address stakeholder engagement in honest and open dialogue about their values and attitudes towards animals (see, for example, the methodology in [60,61]. 

*Continuous Self-critique*

A commitment to continuous self-critique encourages conscientious monitoring of technologies that have animal welfare science at their core. When marrying animal welfare science and ethics with technology, it is key that the information disseminated through these innovations is communicated efficiently to all stakeholders and considers the inclusion and integration of diverging interests, the rethinking of models that already exist, and the facilitation of participative learning and management platforms that incorporate science and values in order to better inform public policies, decision-making and sustainable practices. Continuous self-critique invites the opportunity for collective wading through competing principles that anchor the profession, e.g., non-malevolence, benevolence, and exposure to different systems of thought and discovery to ensure that broader social cum ethical concerns are appreciated and expectations are met. Greater empathy and respect and a deeper understanding of how humans-animals-environmental issues are connected to each other and valued may also emerge. In more concrete terms, tomorrow’s veterinary students should be exposed to the scientific, political, ethical, and social dimensions of animal welfare, so that they can discern between animal advocacy groups that are genuinely committed to promoting animal welfare and more humane and respectful human-animal interactions through sound animal welfare science and groups that have alternative political agendas [96,97]. Minding Animals (https://www.mindinganimals.com/) and Animals and Social Change (https://digitalanimalities.org/upcoming-events/2017/1/24/the-cfhas-conference-event-animals-and-social-change) are examples of conference venues where students and veterinarians can get greater exposure to the dimensions of animal-human interconnections. Animal Welfare Judging and Assessment Contests also provide veterinary students with exposure and training about animal welfare and its multifaceted nature (www.awjac.org). In the US context, participation in professional organizations like the American College of Animal Welfare (ACAW) whose mission it is “to advance animal welfare through education, certification, and scientific investigation” (www.acaw.org), can help promote animal welfare competencies as a significant veterinary specialty. 

## 9. Conclusions

This article discusses the increasing social expectation that veterinarians not only provide good animal care for their patients, but that they also have an ethical responsibility to be exponents of animals and their welfare. In order to meet this expectation veterinarians are: (1) expected, in addition to their traditional role as trusted medical experts, to adopt animal welfare as part of how disease treatment is traditionally understood; (2) challenged to reimagine their professional duties when it comes to disease prevention at the intersection of animal-human-ecosystem health; (3) increasingly forced to develop core competencies in animal welfare in order to provide professional leadership in animal welfare and navigate discourses concerning competing professional priorities and socio-political ideologies and; (4) called to provide feedback on novel networked devices, monitoring technologies and automated animal welfare solutions (e.g., CEMA-AGRI.org CEMA-AGRI.org. (n.d.) Precision Livestock Farming. http://www.cema-agri.org/page/5-precisionlivestock-farming. Accessed 10 June 2018), thus, requiring familiarity with cutting-edge engineering and technological developments in order to critically assess their application to animals’ welfare.

As veterinarians embrace the mantle of being scientific and moral authorities in animal welfare, they will increasingly be invited to utilize their voice more and provide unbiased leadership on issues involving the moral status, treatment and welfare of animals. Doing so will involve being informed by the best science available, and developing greater ethical sensitivity to, and ethical knowledge of, a broad range of ethical issues in clinical practice. Veterinarians should also be better acquainted with technology and have proper training in animal welfare science methods.

Veterinarians should be encouraged to engage in innovating veterinary education and the scientific investigation of animal welfare in their own countries, states, and municipalities, especially in developing regions where the topic of animal welfare is still new and there is much debate about what it might mean in local contexts. Awareness of the current scientific developments within disciplines relevant to animal welfare science, such as animal behavior, evolutionary theory, epigenetics, epidemiology, experimental psychology, social, environmental sciences and so on, together with pedagogical tools, such as problem-solving methods, should be integrated alongside exposure to ethical frameworks (e.g., [98,99,100]), in order to open students’ minds to new possibilities for advancing animals’ welfare. Curricula initiatives targeting animal welfare outcomes should aim for the professional development of veterinarians, so that they are cognizant of the public interest in animal welfare and the public expectation for veterinarians to work with animal welfare in any veterinary discipline, conversing proficiently in multidisciplinary settings and being critical thinkers, as well as practical decision makers [5,7,8,23,24,36,37,38]. Veterinarians who are proficient in science-ethics communication, for example, will likely be considered as more credible interlocutors with different public audiences in their advocacy of animal issues [92]. Through exposure to ethics training and animal welfare science, veterinarians will develop the skills to reflect on and employ ethical thinking that has applications for animals ([7,8,36]; see also https://vet.purdue.edu/CAWS/bioethics/background.php, and http://www.slu.se/vethics) and will be able to provide strong reasoned arguments that reflect the voices of animals and meet societal expectations.
